# Analysis across diverse fish species highlights no conserved transcriptome signature for proactive behaviour

**DOI:** 10.1186/s12864-020-07317-z

**Published:** 2021-01-07

**Authors:** Sonia Rey, Xingkun Jin, Børge Damsgård, Marie-Laure Bégout, Simon Mackenzie

**Affiliations:** 1 Institute of Aquaculture, University of Stirling, Stirlingshire, FK9 4LA UK; 2 Centre for Ecological and Evolutionary Synthesis (CEES), Department of Biosciences, University of Oslo, NO-0316 Oslo, Norway; 3 Institute of Marine Biology, College of Oceanography, Hohai University, Nanjing, 210098 China; 4 Faculty of Biosciences, Fisheries and Economics, UiT The Arctic University of Norway, 9037 Tromsø, Norway; 5 Ifremer, Place Gaby Coll, 17137 L’Houmeau, La Rochelle, France

**Keywords:** Proactive, Animal personality, RNA sequencing, Fish behaviour, Phenotype variation, Convergent evolution

## Abstract

**Background:**

Consistent individual differences in behaviour, known as animal personalities, have been demonstrated within and across species. In fish, studies applying an animal personality approach have been used to resolve variation in physiological and molecular data suggesting a linkage, genotype-phenotype, between behaviour and transcriptome regulation. In this study, using three fish species (zebrafish; *Danio rerio*, Atlantic salmon; *Salmo salar and* European sea bass; *Dicentrarchus labrax*), we firstly address whether personality-specific mRNA transcript abundances are transferrable across distantly-related fish species and secondly whether a proactive transcriptome signature is conserved across all three species.

**Results:**

Previous zebrafish transcriptome data was used as a foundation to produce a curated list of mRNA transcripts related to animal personality across all three species. mRNA transcript copy numbers for selected gene targets show that differential mRNA transcript abundance in the brain appears to be partially conserved across species relative to personality type. Secondly, we performed RNA-Seq using whole brains from *S. salar* and *D. labrax* scoring positively for both behavioural and molecular assays for proactive behaviour. We further enriched this dataset by incorporating a zebrafish brain transcriptome dataset specific to the proactive phenotype. Our results indicate that cross-species molecular signatures related to proactive behaviour are functionally conserved where shared functional pathways suggest that evolutionary convergence may be more important than individual mRNAs.

**Conclusions:**

Our data supports the proposition that highly polygenic clusters of genes, with small additive effects, likely support the underpinning molecular variation related to the animal personalities in the fish used in this study. The polygenic nature of the proactive brain transcriptome across all three species questions the existence of specific molecular signatures for proactive behaviour, at least at the granularity of specific regulatory gene modules, level of genes, gene networks and molecular functions.

**Supplementary Information:**

The online version contains supplementary material available at 10.1186/s12864-020-07317-z.

## Background

Consistent individual differences in behaviour, known as animal personalities, have been demonstrated within and across animal species [1, 2]. Animal personality may provide an adaptive framework to explore the complex interactions between environmental demand and an individual’s capacity to respond [3]. Studies addressing individual variation within a given population, from ecology to genome, have received considerable attention over the past two decades [4–6]. Animal personality (AP) encompasses studies on the consistency of individual response over time and through different contexts including both stressful and non-stressful situations. Réale et al. (2007) [3], within the context of ecology and evolution, proposed five primary animal personality traits (also called temperament traits): (1) shyness-boldness in response to risky situations, (2) exploration or avoidance of new situations, (3) general activity levels, (4) aggressiveness, and (5) sociability. Each of these measured on a sliding scale using a diverse set of methodologies provide data assessing the magnitude and intensity of individual variation and how consistent individuals are over time and across multiple contexts for a given personality trait. It should however be kept in mind that some trait correlations are flexible and can be dissociated during development and modulation of the environment [7]. Some personalities can be related to stress coping styles/behavioural syndromes and vice versa where testing the animals under different stress situations and recording their responses can be effective [8, 9].

Developing tools to reliably identify individuals with contrasting personality traits facilitates the exploration of the underlying molecular and physiological regulation that in turn facilitates efforts to understand adaptation and the evolution of behavioural traits. Significant progress has been made towards our understanding of individual variation within and between behavioural phenotypes and their relationship with transcriptional regulation however major challenges remain [10, 11]. Evolutionary studies using RNA sequencing (RNA-Seq) to address the phenotype-genotype gap have suggested that many genes are transcriptionally linked to a certain phenotype [12]. In fish, studies applying an animal personality approach have been used to resolve variation in physiological and molecular data suggesting a linkage, genotype-phenotype, between behaviour and transcriptome regulation [13, 14]. Such studies provide the background to ask whether convergently evolved traits are the result of convergent molecular mechanisms [11]. The observed convergence of different behaviours across distantly related species suggests that suites of underlying adaptive molecular processes are likely at work [13, 15–17]. Therefore, similar gene expression patterns maybe associated with the expression of convergent phenotypes or indeed distinct regulatory modules may produce an equally functional solution to selective pressures [17]. Transcriptomics provides the ideal platform to interrogate the organisation of the molecular processes underpinning studies in animal behaviour [18–20].

We previously provided evidence that variation in the transcriptome between individuals in a zebrafish (*Danio rerio*) population could be partially resolved by a priori screening for animal personality and this accounted for > 9% of observed variation in the brain transcriptome [14]. Proactive and reactive individuals, fulfilling the 5 traits proposed by Réale and colleagues (2007) [3] within a wild-type population exhibited consistent behavioural responses over time and context that related to underlying differences in regulated gene networks and predicted protein-protein interactions [21]. These differences could be mapped to distinct regions of the brain and provide a foundation toward understanding the coordination of underpinning adaptive molecular events within populations [14, 22]. A major consideration for molecular studies in animal personality is whether the traits described through detailed behavioural analyses are underpinned by large cohorts of genes with small additive effects (polygenic) or discrete sets of gene expression modules conserved across species [21]. Further, both above mechanisms may combine to produce the phenotypes or alternatively there are no shared patterns across species. In this study, we hypothesised that proactive behaviour is a homologous trait, across the three experimental fish species used, underpinned by gene expression networks conserved from a common ancestor. In this study we have taken two distinct approaches firstly, we deployed a targeted approach, a discrete set of mRNAs, using a curated set of genes from our previous study in zebrafish representing differences between proactive and reactive fish [14]. From this curated gene set, we identified mRNAs that were specific to proactive behaviour and quantified their mRNA transcript copy numbers in the brains of Atlantic salmon (*Salmo salar*) and European sea bass (*Dicentrarchus labrax*) screened a priori for personality by using a behavioural test. Secondly, we tested large-scale transcriptome data only for proactive individuals across all three species, all scoring positive for both behavioural and molecular assays, to explore the possibility of a transcriptome signature for proactive behaviour.

## Results

### Behavioural screening for risk taking in groups

From the behavioural screening test performed (response to hypoxia) for both species, *S. salar* and *D. labrax*, a total of 264 proactive and 207 reactive individuals were identified for *S. salar* (Table S1) from all experimental tanks; for *D. labrax* we obtained 120 proactive and 93 reactive individuals. From the total number of individuals screened for behavioural phenotypes, only a subsample of individuals were selected and sampled for whole brain and used for posterior molecular analysis (for *S. salar*: Proactive = 88; Reactive =40 and for *D. labrax:* Proactive = 20; Reactive = 20; Table S1).

### The targeted approach across species

We used individual zebrafish brain transcriptomes derived from a previous study [14] to identify target sequences in both *S. salar* and *D. labrax* that were identified as personality-specific for mRNA abundance scores in wild-type zebrafish. Best BLAST results yielded 3738 and 1734 homologues with high BLAST scores (including high identities, low e-values and high coverage) for sea bass and salmon in respect to the zebrafish data. After manual curation we were able to confidently identify approximately 30 orthologous genes of high quality that could be identified across species (Table S2). Potential targets were selected cloned, sequenced and tested for detectable expression levels in absolute rtqPCR assays (range 10^2^–10^7^ copies). *atpa3* mRNA was identifiable across all 3 species and validated for further analyses. Further mining the data for both function and available expression data QPCR validation studies yielded a total of four targets for use in each species with *atpa3* and *ifrd1* common to both and *cry1* and *ptbp1* specific to salmon and *nedd8* and *gapdh* for sea bass that met our criteria, as described above.

**Fig. 1:**
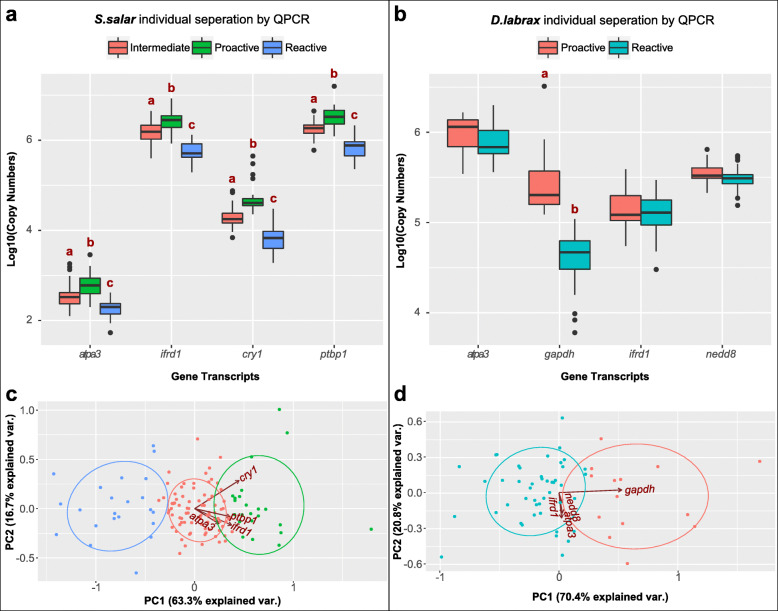
mRNA transcripts that differentiate between personalities in a zebrafish population are transferrable to other fish populations. Log10-transformed copy number of indicated mRNA transcripts grouped by different behaviour phenotypes in (**a**) *S. salar* (**b**) *D. labrax*. Whiskers show standard deviation and different lower-case letters indicate significantly different groups (*p* < 0.001, ANOVA, Tukey-HSD Multiple pair-wise comparisons, 95% confidence level). Principle Component Analysis (PCA) of individual brain with the expression levels of animal personality-specific mRNA transcripts as original variables for (**c**) *S. salar* (**d**) *D. labrax*. The percentage of the variation explained by PC1/2 is shown in coordinates; Dots represent individual brains coloured by behaviour profile, the ellipse represents the core area with the default 68% confidence interval; The arrow represents the original variable, where the direction represents the correlation between the original variable and the PC, and the length represents the contribution of the original variables to the PC. Colour scheme for different behaviour phenotypes is consistent within each species

Individual fish values were clustered (K-means), based on their absolute gene expression values (log^10^ transformed copy numbers) obtained via absolute rtqPCR assays. For *S. salar* optimal clustering size, based upon mRNA copy numbers, was 3, which included proactive, intermediate and reactive. In contrast for *D. labrax*, 2 clusters could be identified as proactive and reactive. Further multiple pairwise comparisons of specific mRNA transcript copy numbers between each behaviour group demonstrated that the three groups in *S. salar* were significantly different for all gene transcripts measured (Fig. 1a). Whilst for *D. labrax*, only GAPDH mRNA copy number was significantly different (Fig. 1b). This was further supported by principal component analysis where *S. salar* individuals could be separated into three clusters with all four detected gene transcripts (Fig. 1c). For *D. labrax,* GAPDH mRNA transcript copy number contributed 70.4% to the grouping of individuals with different behavioural phenotypes (Fig. 1d).

In order to select individuals for the next set of analyses, global transcriptome, we combined our data for both behavioural screening and absolute rtqPCR assays in both species. Our aim, to produce the most accurate dataset possible for RNA-Seq analyses. Individuals confirmed to be proactive by both behaviour- and rtqPCR screening were chosen for subsequent study resulting in 18 proactive individuals for *S. salar* (Figure S1a) and 5 individuals for *D. labrax* (Figure S1b).

### The ‘proactive’ transcriptome approach across species

In total, 12 pair-ended (PE) libraries were constructed via Illumina HiSeq platform. Approximately 10 million PE reads per library past the quality trimming process based on Trim-Galore reports. The final genome- guided de novo assemblies generated by Trinity consisted of 542,302 contigs with an N50 of 1888 bp for *S. salar*, and 189,478 contigs with an N50 of 2784 bp for *D. labrax* (Table S5). Of these two assemblies, 63 and 88% of the total contig sequences were inspected to be “Good” by TransRate for *S. salar* and *D. labrax* (Table S5). According to the criteria of TransRate metrics, a ‘good’ assembly is defined as how contigs are aligned in a way that is consistent with the contig assembly. It has to satisfy all the following conditions (i) where both pair-end reads are aligned; (ii) alignment orientated correctly; (iii) on the same contig; (iv) without overlapping either end of the contig. TransRate: reference-free quality assessment of de novo transcriptome assemblies [23]. As evaluated by BUSCOs, most of the anticipated genes are present as single copies in all vertebrates (BUSCOs), are mostly expressed in both *S. salar* (74%) and *D. labrax* (79%) brain transcriptome assemblies (Figure S2 and Table S4). It should be noted that gene duplication values for *S. salar* are relatively high due to the extra genome duplication event in this species (Table S4). The completeness of reference sequence cDNAs from full-genome annotation is 94 and 87% for *S. salar* and *D. labrax* respectively (Figure S2 and Table S4). Based on the above results (BUSCO and Transrate) we retained both original assemblies generated by trinity without further filtering as a reference for further analysis.

**Fig. 2:**
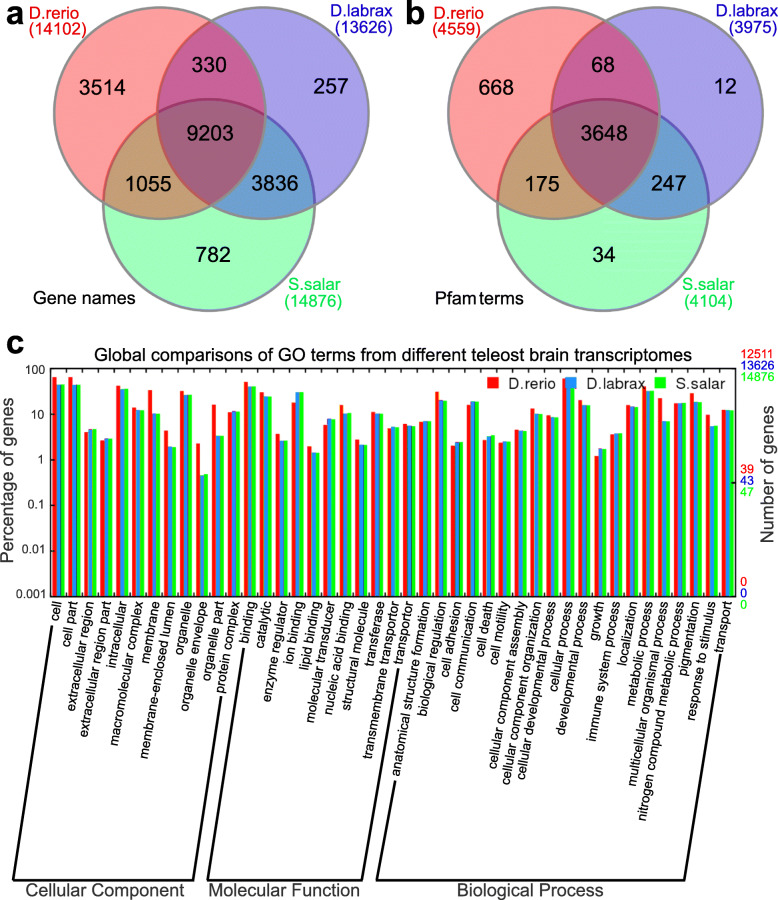
Comparison of three teleost whole brain transcriptomes. UniProt identifiers of all three species were used for comparison and the Pfam (protein family) ID numbers and GO (gene ontology) terms were obtained based on their UniProt identifiers by Biomart (http://www.ensembl.org/biomart) respectively. Venn diagrams are used to visualize overlapping and/or unique **a**) genes and **b**) protein families (Pfam) across the three teleost brain transcriptomes. **c**) histogram shows GO annotations and associated numbers of genes of the brain transcriptomes

In total 22,424 out of 45,220 probes of *D. rerio* microarray were determined to be positive in brains after normalization and background correction (Figure S3 and S4). Subsequently, we combined this data with Illumina Sequencing data from the same tissue [24] to obtain a comprehensive profile of the *D. rerio* brain transcriptome consisting of 14,102 protein-coding genes (Fig. 2a and Table S6). The whole transcriptomic assemblies of *S. salar* and *D. labrax* were mapped to 14,876 and 13,626 protein-coding genes of *D. rerio* UniProt database respectively (Fig. 2a and Table S6). Overlapping analysis confirmed 9203 genes are consistently expressed in brains across all three teleost species (Fig. 2a), which consists of 65.3, 61.9 and 67.5% of the whole transcriptomes from *D. rerio*, *S. salar* and *D. labrax* respectively. Further analysis using conserved protein domains demonstrated that 3648 Pfam modules are shared across all three species, which comprised 80.0, 88.9 and 91.8% from *D. rerio*, *S. salar* and *D. labrax* transcriptomes respectively (Fig. 2b, Table S7). In addition, comparisons of functional GO annotation demonstrated significant similarities across all brain transcriptomes at all three levels, e.g. Cellular Component, Molecular Function and Biological Process, particularly between *S. salar* and *D. labrax* (Fig. 2c).

### In silico analyses of *proactive-related* mRNA abundance

**Fig. 3:**
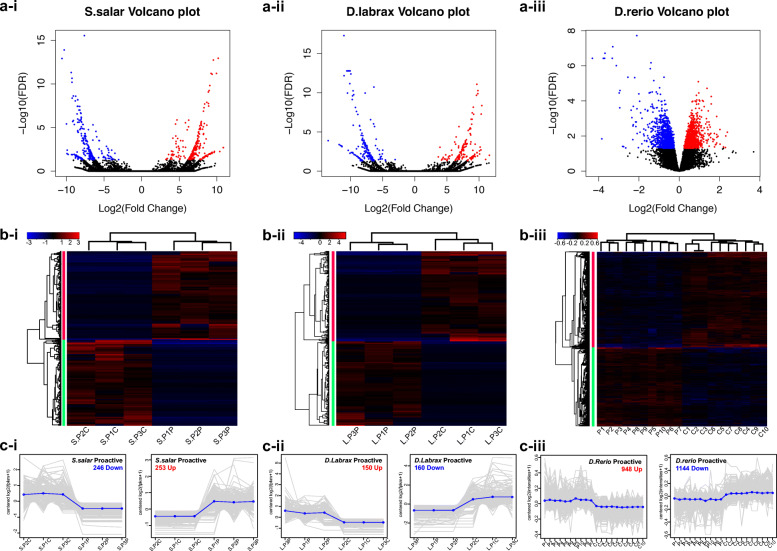
Differential expressed genes (DEGs) in whole brain transcriptomes in proactive *S. salar*, *D. labrax* and *D. rerio*. a-i ~ iii) Volcano plots show DEGs, up-regulated DEGs in the proactive group are shown in red and the control group in blue (log2 (Fold Change)” > 0, and “false discovery rate (FDR)” < 0.05). b-i ~ iii) Hierarchical clustering of DEGs and samples. Heatmaps show the relative expression levels of each DEGs (rows) in each sample (column). Expression values (FPKM for both *S. salar* and *D. labrax* and probe intensities for *D. rerio* are log2-transformed and then median-centred by DEGs. c-i ~ iii) DEG clusters extracted from the hierarchical clustering. X axis: samples; y axis: median-centred log2. Individual DEGs are shown as grey lines; the average expression values per cluster are shown as blue lines. No. of up-regulated DEGs in the proactive group from each cluster is shown in red, control group DEGs are blue. Abbreviations: “S” or “L” are “*S. salar*” or “*D. labrax*” respectively; 2nd letter “P” = “Pool”; Numeric lettering indicates replicates within group; 3rd lettering “P” or “C” is “Proactive” or “Control”)

Firstly, differentially expressed transcripts (DEGs) were identified by comparing “Proactive” and “Control” species (log2FC > 1, FDR < 0.05), in which the latter reference group represented a pool of all behavioural phenotypes for each species (*n* = 18 individuals/species). For *S. salar*, among the total 463,565 transcripts, 253 transcripts (including 245 DEGs) were up-regulated, and 246 (including 236 DEGs) down-regulated (Fig. 3a-i), of these DEGs, 19 were alternative-spliced transcripts from the same gene. For *D. labrax*, with a total of 188,460 transcripts, 150 transcripts (including 138 DEGs) were up-regulated and 160 (including 154 DEGs) down-regulated with respect to control (Fig. 3a-ii) of which 21 DEGs were alternative-spliced transcripts. Finally, for *D. rerio* from a total of 22,424 probes, 948 probes were up-regulated and 1144 down-regulated (Fig. 3a-iii). The *D. rerio* data resulting from microarray analyses (hybridisation) showed a significantly lower dynamic range (fold changes) in comparison to the RNA-Seq data from the other two species, as expected. Hierarchical clustering of DEGs demonstrated relative expression levels of each transcript in either proactive related gene clusters or replicated sample groups to be clustered in each species (Fig. 3.b-i ~ iii). Furthermore, individual transcripts and average expression values of each cluster also highlighted consistent expression patterns within each replicate between proactive and control groups (Fig. 3.c-i ~ iii). Spearman correlation analysis for each replicate within each species demonstrated a positive correlation (> 0.5) for transcripts of “proactive” or “control” groups (Figure S5 and Table S8).

**Fig. 4:**
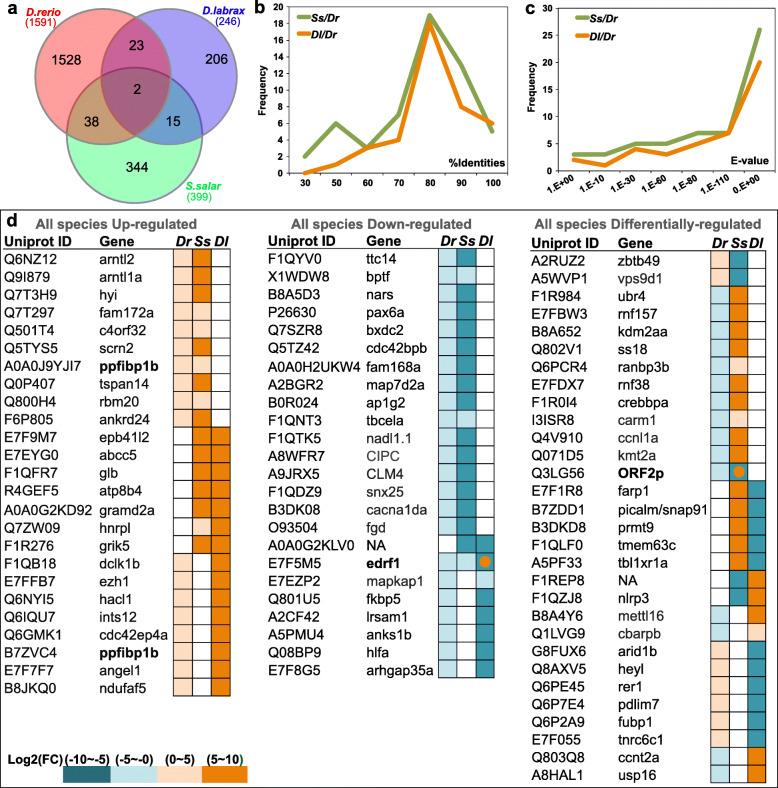
DEGs across proactive brains from three teleost species. **a**) both up- and down- regulated DEG clusters in the proactive group are combined from each species (based upon annotation of gene names in *D. rerio*). Only shared DEGs were kept for further analysis. **b**) Percentage of identities and **c**) e-values for shared DEGs from *S. salar* and *D. labrax* against *D. rerio* UniProt annotations are shown in a frequency distribution to compare sequence similarity and conservation of proactive behaviour related genes. **d**) Shared DEGs across species were subsequently grouped based on the consistency of expression patterns, and colour-labelled for log2 (Fold Change) values

**Fig. 5:**
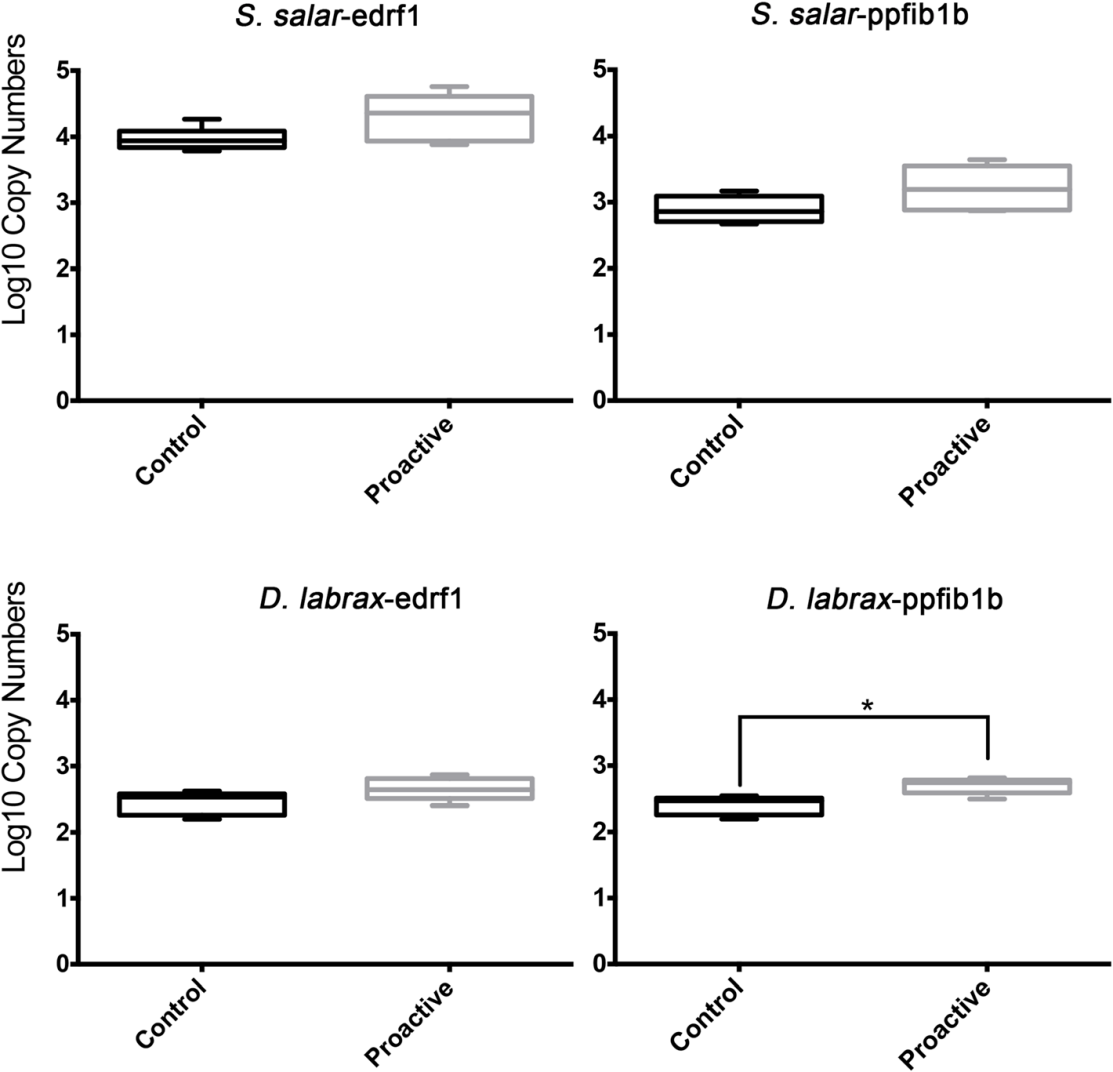
Absolute rtqPCR results of mRNA transcripts specific to proactive behaviour in all three species tested. mRNA copy number is shown for *edrf1* and *ppfibb1p* transcripts in *S salar* (top panel) and *D labrax* (bottom panel) in control and proactive individuals* *p* < 0.05. (N > =5)

In order to facilitate the species-wise comparisons of proactive related DEGs, sequence annotations of the two non-model species were obtained by mapping against *D. rerio* UniProt database using BLASTx. A total of 78 cross-species DEGs (at least expressed in two species) were identified (Fig. 4a and Table S9). Of which, 55 cross-species DEGs were identified in *S. salar* where 85% of DEGs have very high sequence identities (> 60%) with *D. rerio*, and 95% a high confidence E-value (< 1E-10); For *D. labrax,* 40 cross-species DEGs were identified with 97.5% of them sharing high sequence identities (> 60%) with *D. rerio*, and 95% with high E-values (<1E-10) (Fig. 4b and c). Our results indicate that the proactive related mRNAs identified are comparatively conserved across the three species. Further analysis based on expression differences (fold change) of the cross-species DEGs yielded three clusters: i) 24 up-regulated DEGs; ii) 24 down-regulated DEGs; iii) 30 differentially-regulated DEGs (Fig. 4d). Testing targets, both in silico and by absolute rtqPCR, produced two targets that gave detectable transcripts for *S. salar and D. labrax*; *edrf1* (Erythroid Differentiation Regulatory Factor 1) and *ppfibp1b* (Protein-Tyrosine Phosphatase Receptor-Type F: Polypeptide-Interacting Protein-Binding Protein 1). For *edrf1* we did not identify any significant differences by RNA-Seq or QPCR in either species. However, it is worth noting that in mammals, orthologous genes of *edrf1* were found to have alternative splicing features under certain physiological conditions [25], that increase the difficulty to distinguish which particular transcript variant (or variants) is/are correlated to proactive behaviour. For *ppfibp1*, transcript abundance was significantly higher in the proactive group in sea bass (*P* < 0.05) consistent with RNA-Seq data (Fig. 5). A similar tendency, although not significant, was also found for salmon (Fig. 5). The magnitude of differential expression of *ppfibp1* was higher in seabass than salmon when measured by RNA-Seq (Fig. 4d colour scale) but not by QPCR (Fig. 5).

### Functional interactome analysis of risk taking related transcripts across species

**Fig. 6:**
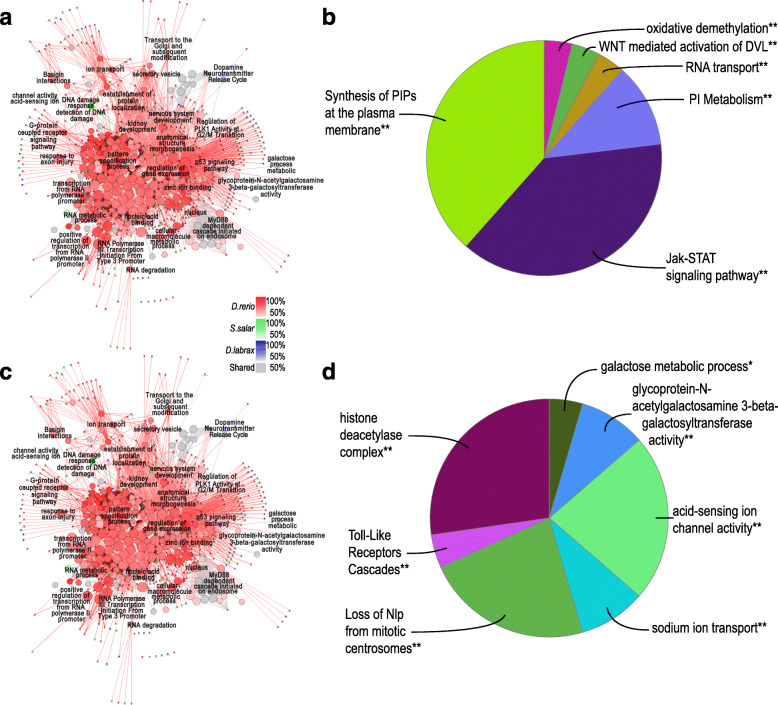
ClueGO network visualization of enriched GO terms and DEGs in brain transcriptomes from *S. salar, D. labrax* and *D. rerio*. Directionally regulated DEGs in proactive phenotypes from each species were analysed expression-pattern-wisely. Enriched/Depleted GOs are identified by a two-sided hypergeometric test with adjusted *p*-values (Benjamini-Hochberg) < 0.05. In each sub-panel: **a**) proactive-up and **c**) proactive-down regulated DEGs and associated GO networks from *D. rerio, S. salar* and *D. labrax* are shown in red, green and blue respectively. The colour gradient shows the gene proportion of each cluster associated with the term. GO terms with < 60% of genes from all three clusters are defined as “shared terms” and represented in grey. **b**) proactive-up and **d**) proactive-down regulated functional groups for shared GO terms across all three species. The name of each group is named using the most significant term in the group. Group sections represent the number of the terms included in each group. *P*-values: “**” < 0.001, 0.001 < = “*” < 0.05

Aiming to understand the functional significance of the correlated transcripts and identify regulatory pathways of proactive behaviour GO network analysis was carried out with all three species datasets (Fig. 6 and Table S10) and without the *D. rerio* dataset (Table S11). Overrepresented GO terms for proactive high abundance DEGs specific GO clusters constituted 22 Groups, of which 6 groups were shared by all three species with the numbers of associated transcripts as follows: PI3K cascade (31), phospholipid (PI) metabolism (41), RNA localization (9), WNT mediated activation of DVL (3), Oxidative demethylation (2) and role of phospholipids in phagocytosis (38) (Fig. 6a, b and Table S10). For proactive low abundance DEGs 29 specific GO clusters were identified, of which 7 groups were shared by all three species including: MyD88 dependent cascade initiated on endosome (35), RNA Polymerase III Transcription Initiation From Type 3 Promoter (5), Regulation of PLK1 Activity at G2/M Transition (17), acid-sensing ion channel activity (37), glycoprotein-N-acetylgalactosamine 3-beta-galactosyltransferase activity (2), peptide N-acetyltransferase activity (17) and ion transport (32) (Fig. 6c, d and Table S10). Numbers of associated genes for each GO term were significantly lower when functional GO analyses when carried out with both ClueGO and CluePedia for *S. salar* and *D. labrax* in comparison with *D rerio* as expected according to DEG input numbers (462 and 284 DEGs respectively). In general, many enriched GO networks shared by all the three species are known to have neurobiological and behavioural significance as we have previously reported e.g. cell division, ATP synthesis, cell adhesion, extracellular matrix remodelling, suggesting that a degree of conserved functionality can be inferred across all three species for the proactive risk-taking phenotype.

## Discussion

Animal behaviour is shaped by the interaction between genes and the environment. The recent rise of evolutionary genomics with its potential to bridge genotype-phenotype gap has led to a diverse cohort of studies (for reviews see: [19, 21, 26]. High-throughput molecular methodologies such as RNA-Seq, addressing the transcriptome, have been deployed in an effort to characterise underlying gene networks or signatures that can be ascribed to particular phenotypic traits aiming to further our understanding of animal behaviour [20, 21]. The transcriptome approach, global mRNA expression patterns in a given tissue or cell population, has the potential to uncover phenotypical patterns however is plagued with significant biological variation. The unsurprising complexity and scale of biological variation at many levels including genotype, development, environment and individual variation within a study population represents a major barrier toward elucidating underpinning mechanisms of animal behaviour [19]. Such complexity may confound the value of molecular data to the evolutionary context of the question where trait variation is likely underpinned by complex polygenic determination [27].

In this study we deployed behavioural and molecular assays to explore two outstanding questions. Firstly, is the variation in specific brain mRNA transcripts related to different personalities in one fish species (wild-type zebrafish) conserved across distantly related species? and secondly, is there a conserved whole brain transcriptome signature for proactive behaviour within the *Teleostei* that are separated by significant evolutionary time?

Recognising individual variation within and across species has been brought to the fore by the field of animal personality/behavioural syndromes [6]. Such studies based upon consistency over time of specific individual behavioural traits such as risk taking, aggression and activity address the adaptive value of an individual’s response during environmental adaptation [2]. A major aim is to elucidate the relationship between behaviour and state of an individual in order to understand the evolutionary significance of consistent variation within species and across species [6]. In fish, animal personality and stress coping styles have been used to resolve variation in characterising underpinning regulatory physiological and molecular mechanisms that provide critical insight into adaptive evolutionary processes [14, 28–31]*.* In this study, a common behavioural test was designed for both species in order to harmonize the AP screening and obtain the same behavioural outputs regardless of the species. The hypoxia test was used as for personality screening and was developed and deployed in captive populations of *S. salar* [32] and *D. labrax* [33] providing biological samples characterised for animal personalities. The hypoxia test had been previously tested, and validated by physiological stress markers like cortisol and monoamines, as a personality screening test for rainbow trout, *O. mykiss* [34] and for European Sea bass [35]. The same hypoxia test and stress marker evaluations were further validated for *S. salar* [32, 33, 35] in similar studies. Hypoxia is a well-studied physiological phenomena that commonly occurs in natural and confined habitats, such as aquaculture production, with significant implications for optimal physiological function [36]. Hypoxic conditions promote rapid changes in fish favouring escape behaviour particularly under severe hypoxic conditions [37] emulating risk taking proactive behaviour. Therefore, the intensity of O_2_ deprivation and its relationship with the underlying species-specific physiology may influence the selectivity of the test particularly to differentiate intermediate and reactive personality types [38]. Understanding the eco-physiological constraints in the design and development of high-resolution behavioural screening protocols for different species and their associated personalities with emphasis upon oxygen, temperature and light remains a challenge for comparative studies.

Using a zebrafish consensus brain transcriptome correlated to a proactive or reactive personality [14] we identified a set of mRNA transcripts across public genomic resources for both *S. salar* and *D. labrax* (> 30/species). Our in silico approach, based upon previous studies [39], highlights both the value of current genomic resources for fish particularly aquaculture species and the remarkable gaps in knowledge remaining. This was patent in our analyses concerning gene function in non-model species as was highlighted by the low number of ‘common’ transcripts we were able to identify across fish species. We were able to validate observed personality-specific variation in four target mRNAs for *S. salar* and 1 for *D. labrax* out of a total of 30 absolute rtqPCR assays carried out for each species. As all experimental samples were taken from non-stressed baseline populations the complex variation observed, for example reference mRNAs such as *gapdh* [40], particularly for *S. salar* are indeed surprising and highlight the pitfalls of using reference mRNAs. Importantly, our PCA analysis was able to discriminate for all three distinct AP groups of *S. salar* in the measured population. Interestingly, in stress coping style studies in fish where a challenge is applied, a higher variation in specific mRNA transcripts is measured across the control population of individuals representing all APs and such variation is then reduced during the adaptive response [28–30]. It is worthy to note that there is less observed variation in particular mRNAs between proactive individuals than between any other measured AP grouping in *D. rerio* [14, 28]. Such observation suggests the possibility of retrofitting data using personality-specific threshold mRNA abundances to explore individual differences in the huge array of existent experimentation and is an intriguing prospect for future studies.

Secondly, we applied RNA-Seq to individuals, *S. salar* and *D. labrax*, that scored positively for proactive traits in both behavioural screening and absolute rtqPCR. Using whole brain preparations, we hypothesized that if a transcriptome signature for a proactive brain in fish is indeed selectively conserved across species such granularity would be acceptable. In this case the most recent ancestor for all three species is placed 230 million years ago [41, 42]. As in many studies we used a static comparison that also has associated caveats that should be considered in context [43]. Our data revealed strong correlations between brain transcriptomes and the proactive personality in each of the species. These data suggest that a molecular basis for neuro-genomic networks underpinning proactive individuals exists across all tested three teleost species (PCA analysis, Supporting Fig. 5 and Fig. 6). However, a relatively small number of DEGs are shared across species for the proactive phenotype, suggesting that the genetic basis for proactive behaviour is diverse at the level of brain mRNA population. Similar results have been reported in other species indicating that divergent adaptive pressure (ecological niche) shaped the evolution of such phenotypical traits and the underpinning regulatory networks are likely highly varied thus hampering attempts to harmonise the molecular basis of regulation [18]. Our data highlights, through GO enrichment analyses, that multiple core functions across the three species are indeed conserved in the proactive brain although a set of specific gene-based circuits common to all cannot be identified (Table S10). Furthermore, specific rtqPCR assays for shared targets did not provide conclusive evidence of single gene effects. Significant effects in GO enrichment categories, with > 10 contributing genes common to all three species, highlighted cell signalling systems, metabolism, cell cycle and ion transport as divergent for proactive individuals. Such divergence in neurobiological and behavioural phenotypes are modelled through multiple molecular levels (transcriptomic, proteomic and epigenomic) on the basis of physical interactions and correlations within and across multiple molecular levels [43]. In order to discern specific gene modules, if indeed they are present, will require further studies at increased resolution.

## Conclusions

Our data supports the proposition that highly polygenic clusters of genes, with small additive effects, likely support the underpinning molecular variation observed in proactive behaviour in fish rather than a limited number of gene modules with large effects. Although the limitations of our study may have impacted upon our capacity to identify specific genes in our opinion it is unlikely that deeper sequencing would identify significantly more interactions. As previously mentioned the proactive phenotype itself is characterised by a lower level of mRNA variation between proactive individuals in both carp and zebrafish [14, 28]. Observed inter-individual variation in zebrafish is significantly higher between reactive individuals [14] potentially highlighting the difficulties in designing behavioural assays to characterise the complexities of the reactive personality. Our results support a role for convergent evolution for animal personalities across the *Teleostei* as observed in the results from the behavioural assays deployed.

Some studies have reported that similar sets of genes are often associated with the expression of convergent phenotypes [17] and homology at the level of genes, gene networks and molecular functions occurs despite differences at other mechanistic levels [44]. However, the polygenic nature of the proactive phenotype herein measured questions the existence of specific molecular signatures for proactive behaviour, at least at a granularity of specific regulatory gene modules. The observed variation in our system highlights the challenges ahead toward understanding complex behaviour at the molecular scale.

## Methods

### Fish Behavioural screening

Behavioural screening was used to select for animal personality (AP) for both European sea bass, *Dicentrarchus labrax,* and Atlantic salmon*, Salmo salar.* Screenings were conducted following methods described in (Ferrari et al., 2015) [33] for *D. labrax* and a similar method with modification for *S. salar* (Damsgard et al., 2019) [32].

#### *Salmo salar*

Briefly, the study was conducted at the Aquaculture Research Station in Tromsø, northern Norway, using age 0+ Atlantic salmon (Atlantic QTL-innova IPN). The fish were reared at 10 °C, in a continuous 24 h light regime and surplus feeding (Skretting Nutra). Fish were individually tagged using internal 12 mm PIT-Tags (HPT12 tags, Biomark, Boise, US), injected with a MK-25 implant gun. The fish population (*n* = 480, divided over 8 groups) was reared in circular holding tanks (~ 116 L) with flow through fresh water. Average weight 2 weeks prior to the experiment was 57.1 ± 7.3 g. Hypoxia sorting consisted of two custom-made circular tanks (~ 200 L, diameter 65 cm, water depth 60 cm, Cipax AS, Bjørkelangen, Norway), i.e. hypoxia and normoxia tanks. The tanks were connected at the surface level by a tube (inner diameter 9 cm), containing an integrated custom-made spool PIT-Tag antenna (Biomark Ltd., Boise, US), linked to a Biomark FS2001 reader. Tag manager software was used to identify fish leaving to the normoxia tank and those staying (independent of the decreasing oxygen level). Each tank had a separate water inlet and outlet. In the hypoxia tank the inlet was connected to a N_2_ gas exchanger (15 mg N_2_ L^− 1^), which deoxygenated incoming water. Oxygen levels (mgL^− 1^) in the tanks were monitored every minute, using an O_2_-monitoring system (Loligo Systems, Tjele, Denmark). Control tests prior to the experiment demonstrated that oxygen depletion was homogenous throughout the water column. Two video cameras were mounted outside the tube on each side of the opening and over water, in order to monitor fish movement. Each test had a duration of approximately 5 h starting at 8.30 AM and all tests were conducted in an equal manner. Prior to the test, the tanks were cleaned, water temperature regulated if necessary and the water flow in each tank set to 3.5 L min^− 1^. Fish were transferred to the experimental set-up as carefully as possible and released into the hypoxia tank. The two tanks were left undisturbed behind an opaque curtain for the duration of the trial. The monitoring of the fish started immediately, and the fish were allowed to acclimate for 2 h prior to the change in the oxygen level. During the decline in oxygen, the water flow in the hypoxia tank was directed through the gas exchanger, and a door between the hypoxic tank and the normoxic tank was opened so fish could freely swim between the tanks. After the oxygen level in the hypoxia tank reached 25% O_2_ saturation, the screening was terminated and the fish transferred back to their holding tanks. Fish that left the hypoxia tank were classified as leavers and considered proactives. Fish that never left the tank were considered stayers and classified as reactives. All fish was sampled for blood plasma cortisol to confirm the activity levels of the HPI axis corresponding to proactive and reactive personalities. (see [32] for details). After sorting, fish were left undisturbed in their holding tanks for a period of two and a half months, before sampling at basal conditions. Proactive and reactive individuals were sampled directly from holding tanks. Immediately after collection, individuals were euthanized with a lethal overdose of 1 g/L MS-222 (Finquel®, Argent Chemical Laboratories, Redmond, WA, USA). All fish were rapidly weighed and fork length measured. Whole brains were dissected out and placed in individually labelled tubes and stored at − 80 °C for analysis of gene expression.

#### *Dicentrarchus labrax*

Fish were hatched and reared at the experimental research station; Ifremer (Palavas-les-Flots, France) according to seabass rearing standards [45]. Three triplicates of 120 fish (60 per tank) were used and each triplicate was housed in a 1.5 m^3^ tank in a sand filtered open flow system. Three batches of 120 individuals were screened at 215 days post hatching (dph) for hypoxia tolerance [33, 34] in order to assign animal personality (AP) and then returned to original 1.5 m^3^ tanks without modifying group composition. For the hypoxia test, oxygen concentration was decreased in one chamber of a two-chamber tank and escape from the hypoxic to the normoxic chamber was assessed. Experiments were carried out using two identical circular tanks (70 l, h: 48 cm, diameter: 49.5 cm,) attached via a transparent acrylic pipe (diameter: 11 cm, length: 30 cm, height from bottom: 23 cm) see [46]. Each tank was considered to be a separate environment and was equipped with an independent oxygen and air supply that were switched off during trials. Sixty fish were placed in one chamber of the tank, subsequently the hypoxia tank, and were allowed to acclimate to the conditions for 30 min. To induce hypoxic conditions nitrogen bubbling was used to decrease oxygen saturation from 90 to 8% in 1 h. The second chamber of the tank was maintained under normal conditions. Once an individual escaped from the hypoxic tank into the normoxic tank it was immediately netted and placed in a separate tank before being anesthetized, benzocaine 200 ppm, and tagged with 12 mm ISO PIT tags, measured for weight and returned to their respective tanks. Assignment of AP categories was determined as follows. The first 20 fish escaping hypoxic conditions were proactive (P), the ~ 20 followers were intermediate (I) and the remaining fish, with no escape behaviour, were reactive (R). The hypoxia test ended when two thirds of the fish had escaped from the hypoxia tank or when 8% oxygen saturation was reached (water temperature 20 °C, salinity 26.9). This operation was repeated for all experimental fish. SCS assignation in each tank was: Tank 1: 40 P, 39 I and 40 R, Tank 2: 40 P, 61 I and 19 R and finally Tank 3: 40 P, 46 I and 34 R. Fish were sampled at 342 dph (mean weight 89.2 ± 31.8 g) with prior 24 h fasting. Fish were anaesthetized in their home tank using benzocaine, 200 ppm, and gathered in a smaller holding tank. PIT tags were read, weights were recorded, and fish were separated into new tanks according to AP. A random subsample of screened fish were immediately sacrificed using an overdose of anaesthetic (benzocaine) and kept on ice for further dissection. Whole brains were extracted and immediately frozen with liquid nitrogen and stored at − 80 °C. (see [33] for details).

### qPCR analysis of fish brains

#### RNA isolation and cDNA synthesis

Individual brain samples were separately weighed and homogenized with Tri-Reagent following manufacturer’s instructions (1 mL/100 mg of tissue; Molecular Research Centre, Sigma-Aldrich, UK). Total RNA was extracted from individual fish brains for both *S. salar* (Proactive = 88; Reactive =40, Figure S1a and Table S1) and *D. labrax* (Proactive = 20; Reactive = 20. Figure S1b and Table S1) using the standard TriReagent (Sigma-Aldrich, UK) based method following manufacturer’s instructions. The concentration of each sample (total RNA) was quantified by Nanodrop (ND-1000) and quality visualized under UV light in a 1% agarose gel containing 1 μg/ml ethidium bromide. 2 μg of total RNA was taken from each individual to synthesize cDNA with SuperScript III RNase Transcriptase (Invitrogen) and oligo-dT primer (Promega).

#### Identification of salmon and sea bass target mRNAs using the zebrafish AP transcriptome

AP-specific gene lists were derived from our previous study in zebrafish [14] using individual zebrafish brain transcriptomes (dataset http://www.ncbi.nlm.nih.gov/geo/; GEO Accession: GSE40615GEO). Briefly, in this study behavioural tests including risk-taking, activity, mirror image stimulation and latency to feeding after confinement were carried out on a population of 280 zebrafish from a wild-type background. A sub-set of individuals exhibiting consistent AP were selected for brain transcriptome analyses, *n* = 10, for each animal personality. Microarray hybridisations were performed using the Zebrafish V2 (G2519F) 4x44K Agilent oligonucleotide microarray following standard methods according to manufacturer’s instructions (Agilent Technologies). To identify DEGs for each personality we selected genes that were highly significant (ANOVA *P* < 0.001) for proactive and reactive individuals. All *p*-values were adjusted with a false-discovery rate (FDR) correction for multiple testing by the Benjamini–Hochberg method (Benjamini & Hochberg 1995). All genes with FDR-corrected p-values < 0.05 were considered significant.

In silico cloning in the target species was carried out using genomic resources. These resources were: A all available public resources; *S. salar* were derived from the NCBI (994,195 sequences) and UniProt (> 9 K sequences). B All public resources for sea bass (*Dicentrarchus labrax*) UniProt (> 2.5 K sequences) and NCBI (86,801 ESTs) were added to sequence collections for sea bass held in the AquaSea server which forms part of the Aquagenomics website (http://www.aquagenomics.es). Sea bass resources were released in 2015. In order to maximize identification of salmon and sea bass mRNAs we applied our best BLAST iterative methodology [28, 39] (Goetz et al., 2010; Mackenzie et al., 2009). The scripts used consist of a four-step iterative BLAST, combining BLASTx: BLASTn: BLASTx: BLASTn, until a best hit description is assigned, the two first rounds are based on best description and the two next rounds based on best BLAST scores. If no description is found after the 4 rounds, sequences appear as “no hit found”. The e-value cut-off was set to 1E^− 20^ and the best BLAST hit with both highest similarity and coverage and lowest e-value was assigned as the mRNA transcript identity. BLAST results obtained for each species filtering at <1E-20 are available as Supplementary material (Table S2).

#### Curation of results

The lists obtained were then manually filtered to remove redundancy, cross check annotations and curated using the following criteria: 1) Species specificity, 2) contig length, 3) lowest e-value and 4) identity score. We found that BLASTn results were adequate for the analyses. A manual search for functional significance was undertaken using both classical literature search methods and also database orientated searches for functionality in model organisms for example GeneCards. This was also supported by network building in the Cytoscape platform aiming to identify interactions between the mRNAs identified.

#### Absolute qRT-PCR

All primers for QPCR were design by BatchPrimer3 v1.0 based on different sources of template genes as: i) for individual separation of mRNA transcript abundance target marker genes were chosen from the proactive-related gene list based on previous study on *D. rerio* [14] primers were designed based on the orthologous sequences from *S. salar* and *D. labrax* respectively (Table S2). Target genes were validated for each species using thermal gradient RT-PCR and the products that met the quality criteria were cloned into bacterial plasmids. ii) for the verification of RNA-Seq results, sequences of overlapping proactive-related mRNA transcripts (*ppfibp1b* and *edrf1*) across all three species were obtained from either the transcriptome assemblies (for *S. salar* and *D. labrax*) or from the UniProt database via unique identifiers (*D. rerio*) (Table S2). cDNA (2 μl) was used as a template for PCR with gene-specific primers. Target mRNAs were amplified using MyTaq HS DNA Polymerases (Bioline, UK), and amplicons were separated on 1% agarose gels, stained with ethidium bromide and purified with NucleoSpin® Gel and PCR Clean-up (MACHEREY-NAGEL, Germany). Purified PCR products were ligated in pGEM-T easy vector (Promega, USA) and transformed into *E. coli* (DH5a strain). One selected transformant of each construct was grown to obtain plasmid DNA (Miniprep kit, Macherey-Nagel). All constructs were verified by Sanger sequencing (GATC Biotech).

Absolute quantification was performed and the copy number of each transcript, derived from the standard dilution curve obtained from target plasmids was analyzed using a Thermocycler Stratagene Mx3005P (Agilent, USA). Each sample was tested in triplicate in a 96-well plate. The reaction mix (20 μl final volume) consisted of 10 μ of SYBR Green mix (Aligent, USA), 0.5 μl of each primer (20 μM), 7 μl of H2O and 2 μl of a 1/10 dilution of the cDNA sample. The thermocycling program consisted of one hold at 95 °C for 3 min, followed by three-step 35 cycles of 15 s at 95 °C, 10 s at 58 °C and 10 s at 72 °C. No template controls (NTCs) were used to assure no false positive signals were calculated.

#### Statistical analysis for behavioural and QPCR data

Exploratory analysis of the gene expression data in relation to the behavioural data was performed with specific software (AutoDiscovery®, Butler Scientifics). The approach evaluates Spearman’s Rank correlation coefficients for every pair of numerical variables and one-way ANOVAs for every pair of qualitative numerical variables within the consolidated data set in order to extract the most relevant relationships between the variables (Exploratory Data Analysis or EDA process). This was used as an exploratory analysis without significance corrections for false discovery rate (FDR) as the main objective was to suggest potential associations between multiple variables to be further explored. Correlations between all mRNAs, gene expression, tank, sex and weight were also performed. Data was tested for normality with a Kolmogorov-Smirnoff test and Levene’s test for homogeneity of variances. Non-normal data was log_10_ (var + 1) transformed. Tank effects on fish population total weight and gene copy number were also checked either with paired samples T-test or ANOVA tests. Post-hoc Scheffé or Dunnett test, for non-homogeneous variances, were performed for specific significances. A GLM ANCOVA was used to test for significant differences in gene expression between all individuals screened for AP in the populations studied. Dependent variable was Log copy number for each target gene with AP as the fixed factor. Tank and weight were used as co-variables.

To use the individual mRNA transcript abundance data to screen for AP we firstly analysed individuals using the K-mean cluster method, based on absolute mRNA transcript levels for each individual for both species. Optimal cluster numbers were chosen and different clusters were named following the cluster groupings. The criteria of classification was based on our previous data for zebrafish behavioural phenotypes. As a second approach we applied statistical comparisons between different personalities of both species following one-way ANOVA (individual grouping) or Two-way T-test. Significance was reported at *P* < 0.05. All analyses were performed with SPSS v19 (IBM®). Graphs were plotted using Prism5® for MacOS X, SPSSv19® or EXCEL® for Mac 2011 (v14.4.6).

The statistical difference of mean expression levels of gene markers among behavioural groups were calculated by R function “aov” (ANOVA) together with post hoc multiple pairwise comparisons by “TukeyHSD”. Significantly different groups were reported by LSD.test (R package “agricolae”) at *p* < 0.05. The Principle Component Analysis (PCA) of individual fish brains was performed by R function “prcomp” without normalization and scale.

#### cDNA libraries preparation and RNA seq

Only individuals tested to be proactive with coherence in both behavioural screening and absolute QPCR assays were classified as proactive. For control reference groups, individual RNA samples were pooled proportionally following 25% proactive, 25% reactive and 50% intermediate for both species. In total, 12 pools of total RNA were prepared; 3 Proactive pooled samples and 3 control pooled samples for both species (Table S3). All pooled RNA samples were quantified with Nanodrop (ND-1000) and a Qubit Fluorometer (Life Technology) respectively. The RNA integrity and quality were also assessed with a Bioanalyzer 2100 (Agilent Technologies), of which only samples with a RIN > 8 were further processed (Table S3). Total RNA (1 μg) for each pooled RNA sample was used for reverse transcription by SuperScript III RNase Transcriptase (Invitrogen) following the manufacturer’s protocol, with the only exception of reduced RNA fragmentation time to maximize obtention of longer reads [47]. The cDNA libraries were constructed by TruSeq V2 kits (Illumina, CA, USA) and sequenced on a Illumina HiSeq 2500 platform at the Norwegian Sequencing Centre (raw reads: https://www.ncbi.nlm.nih.gov/bioproject/PRJNA543167; sequence depth approx.: 20 × 10^6^ reads/library). The adapters (indexers) in the paired-end raw reads were trimmed out by the quality control tool Trim-Galore (a wrapper tool based on Cutadapt Version 1.4.2 and FastQC Version 0.10.1) for high throughput sequence data, as set up by default quality threshold of Q20. The FastQC reports from both before- and after-trimming were checked.

#### Genome guided de novo transcriptomic assembly and completeness estimation

To facilitate downstream analyses, the after-trimmed reads across all samples (both proactive and control) were concatenated together into a single dataset for follow-up assembly for *S. salar* and *D. labrax* respectively. Trinity was used to generate a de novo transcriptomic assembly. For each species, all ‘left’ reads were combined into a single file, and the same was applied for the ‘right’ reads. Trinity parameters were held as default (Version 2.0.6) [48] with the “genome_guided_max_intron” parameter set to 15,000 for *S. salar* and 10,000 for *D. labrax*. The reference genome sequence for *S. salar* was obtained from NCBI (Accession No.GCA_000233375.4, assembly ICSASG_v2) and *D. labrax* reference genome was obtained from the online database (http://seabass.mpipz.mpg.de).

Quantitative assessment of trinity assemblies for both *S. salar* and *D. labrax* assemblies were measured based on evolutionarily informed expectations of gene content from near-universal single-copy orthologs selected from OrthoDB by BUSCO (v1.1B1) (python3/3.5.0; gcc/5.2.0; emboss/6.5.7; hmmer/3.1b1). Additionally, assemblies were inspected by Translate (1.0.2) which assigned scores based on alignments of reads to assemblies (Figure S2 and Table S4).

#### Whole brain transcriptomic annotation and species-wide comparisons

For *D. rerio*, microarray data (Agilent V2, G2519F, 4x44K) [14] were re-analyzed as follows: the probe sequences were extracted, and the sequence annotation updated using both BLASTn (2.2.26) against RefSeq (GCF_000002035.5_GRCz10, 54,437 sequences) and BLASTx (2.2.26) against UniProt (GRCz10, 59,208 sequences). After background correction (control and low expressed probes removed) and normalization by R (3.2.2) package “limma” (3.24.15) only probes determined to be positively expressed (N > =4 arrays) in both control and proactive groups were retained for further analysis (Figure S3). To further complement the *D. rerio* transcriptome information the RNA-Seq dataset (GSE61108) [49] (Figure S4) was parsed into the *D. rerio* transcriptome increase and improve comparability with the brain transcriptomes of *S. salar* and *D. labrax* obtained on the same Illumina platform. To facilitate species-wide transcriptome comparison the gene identifiers from the two non-model species were substituted for zebrafish identifiers based on sequence similarity. For *S. salar* and *D. labrax*, both transcriptome assemblies were compared to the *D. rerio* UniProt database (GRCz10, 59,208 sequences) using BLASTx (2.2.26). The UniProt identifiers of all three species were used for the following comparisons: i) “gene names”, “Pfam ID numbers” and “GO (gene ontology) terms” were obtained based on their UniProt identifiers by Biomart (http://www.ensembl.org/biomart) respectively; ii) Venn diagram are used to visualized overlapping and/or unique genes and protein families (Pfam) across three teleost brain transcriptomes. iii) Functional GO terms classification was calculated by online software WEGO.

#### Proactive-related gene identification and cross species comparison

Gene expression changes between proactive and control groups were compared using two distinct methods for RNA-Seq, e.g. Agilent microarray hybridization and Illumina sequencing. For the first approach, the intensity value of hybridization for each probe across all *D. rerio* samples (*N* = 20) was measured by R package “limma”. For RNA-Seq the TMM-trimmed (trimmed mean of M values) FPKM (Fragments Per Kilobase of exon per Million reads) value of each transcript was calculated for samples (*N* = 6) for both *S. salar* and *D. labrax* using “edgeR”. For *D. rerio*, the differential expressed genes (DEGs) were calculated and compared, with threshold of FDR < 0.05 and log_2_“fold change” > 0 (*N* = 10 for each group). For *S. salar* and *D. labrax*, the expression abundance of transcripts were estimated as FPKM which were calculated by RSEM [50] and normalized by TMM. Finally a set of pair-wised differential expression analysis were conducted separately using the R package “edgeR” for both species following Trinity DEGs identification pipeline [48] with the same statistical threshold as that of *D. rerio*, i.e. FDR < 0.05 and log_2_ “fold change” > 0 (*N* = 3). In order to estimate sequence conservation and the consistency of expression patterns in each species the following comparisons were conducted. The numbers of overlapping “Gene names” of proactive-related DEGs from each species were compared and visualized using a venn diagram. Only those expressed in at least 2 species were labelled as conserved proactive-related mRNAs and were kept for the subsequent analysis. The BLASTp results (Identities % and E-values) of these highly conservative DEGs from both *S. salar* and *D. labrax* against *D. rerio* were analysed respectively and then log_2_-transformed fold changes of these highly conserved DEGs were extracted for comparison.

#### Functional gene ontology enrichment and network analysis

Functional gene ontology enrichment and network analysis were conducted using Cytoscape (v3.3.0) plugin ClueGO and CluePedia (v2.2.3) following standard pipelines [51]. Briefly, proactive up- or down-regulated DEGs were separately analysed using the model species mode for *D. rerio* (Taxonomy ID:7955) for all three species. Numbers of associated genes for each GO term were setup differently according to the number of DEGs identified in each species (1% for *D. labrax* with 284 DEGs, 2% for *S. salar* with 462 DEGs and 7% for *D. rerio* with 2078 DEGs). GO levels from 3 to 20 were examined with the “GO Fusion” option chosen. Enriched/Depleted GOs were identified by two-sided hypergeometric test and with only adjusted Mid *P*-values (Benjamini-Hochberg) less than 0.05 being kept. The percentage for a Cluster to be significant was set at 60%. The name of each functional group (Overview term) was given by the leading term with the smallest P-value in the group. The network grouping Kappa Score threshold was 0.30. The GO annotation data base versions were: KEGG (15.01.2016, 5461), REACTOME (15.01.2016, 6173), GO_CellularComponent-Custom (08.01.2016_00h21,14,997), GO_MolecularFunction-Custom (08.01.2016_00h21, 15,812), GO_BiologicalProcess-Custom (08.01.2016_00h21, 15,294) and InterPro_ProteinDomains (21.03.2016, 14,291).

## Supplementary Information

**Additional file 1: Table S1.** Individual Running number by behavioural and QPCR analyses

**Additional file 2: Table S2.** BLASTn results and primers for ALL QPCR (XLS 1079 kb)

**Additional file 3: Table S3.** RNA samples pooling and bioanalyser report.xlsx

**Additional file 4: Table S4.** Completeness assessment of transcriptome assembly by BUSCO and Transrate (XLS 23 kb)

**Additional file 5: Table S5.** Trinity assembly statistics

**Additional file 6: Table S6.** Global brain transcriptome comparisons genelist (XLS 1736 kb)

**Additional file 7; Table S7.** Global brain transcriptome comparisons Pfam (XLS 531 kb)

**Additional file 8: Table S8.** DEG Spearman Correlation matrix (XLS 20 kb)

**Additional file 9: Table S9.** DEG list logFC & FDR

**Additional file 10: Table S10.** Shared by 3x Species Functional Groups with Genes (XLS 24 kb)

**Additional file 11: Table S11.** Shared by 2x Species Functional Groups with Genes (XLS 16 kb)

**Additional file 12: Supporting Fig. 1.** Individual personality identification using both behaviour test and rtqPCR assays. Unique individual running numbers were used to identify proactive individuals scoring positive for boldness and gene expression. a) *S. salar* proactive individuals *N* = 18; b) *D. labrax* proactive individuals *N* = 5. **Supporting Fig. 2.** Completeness estimation of Trinity assemblies of Illumina RNA-Seq by BUSCO. Abbreviation: Ss: *S. salar*; Dl: *D. labrax*; Trans: transcriptome; Geno: Genome. **Supporting Fig. 3.** Microarray normalization of *D. rerio* brain transcriptome. a) Distribution densities of probe intensities were compared before- (a-i) and after (a-ii) normalization; b) Log2-transformed intensities of each microarray were shown before- (b-i) and after (b-ii) normalization. **Supporting Fig. 4.** Transcriptome gene list of *D. rerio* brain with annotations. The numbers of annotated genes obtained from [14] are shown in orange; Illumina RNA-Seq sourced annotations [24] are in Dark Green. **Supporting Fig. 5.** Spearman Correlation estimation of DEGs within each species sequenced by Illumina platform. Heat map showing the hierarchically clustered Spearman correlation matrix resulting from comparing the transcript expression values (TMM-normalized FPKM) for each pair of samples from both species: i.e. a) *S. salar*; b) *D. labrax*.

## Data Availability

Transcriptomic microarray data: GEO; http://www.ncbi.nlm.nih.gov/geo/; GEO Accession: GSE40615; Sequence Read Archive: SRA; https://www.ncbi.nlm.nih.gov/bioproject/; BioProject Accession: PRJNA543167; (SRA accession SRR9089153-SRR9089164).

## Abbreviations

AP: Animal personality
dph: Days post hatching
P: Proactive
I: Intermediate
R: Reactive
PIT: Passive integrated transponder
FDR: False discovery rate
DEG: Differentially expressed genes
PE: Pair-ended
GO: Gene ontology
