# Supplementary material for: Analysis across diverse fish species highlights no conserved transcriptome signature for proactive behaviour
Source: BMC Genomics. 2021 Jan 7;22:33. doi: 10.1186/s12864-020-07317-z (PMC7792025; doi:10.1186/s12864-020-07317-z)
Supplement: Supplementary file 12 — Additional file 12: Supporting Fig. 1. Individual personality identification using both behaviour test and rtqPCR assays. Unique individual running numbers were used to identify proactive individuals scoring positive for boldness and gene expression. a) S. salar proactive individuals N = 18; b) D. labrax proactive individuals N = 5. Supporting Fig. 2. Completeness estimation of Trinity assemblies of Illumina RNA-Seq by BUSCO. Abbreviation: Ss: S. salar; Dl: D. labrax; Trans: transcriptome; Geno: Genome. Supporting Fig. 3. Microarray normalization of D. rerio brain transcriptome. a) Distribution densities of probe intensities were compared before- (a-i) and after (a-ii) normalization; b) Log2-transformed intensities of each microarray were shown before- (b-i) and after (b-ii) normalization. Supporting Fig. 4. Transcriptome gene list of D. rerio brain with annotations. The numbers of annotated genes obtained from [14] are shown in orange; Illumina RNA-Seq sourced annotations [24] are in Dark Green. Supporting Fig. 5. Spearman Correlation estimation of DEGs within each species sequenced by Illumina platform. Heat map showing the hierarchically clustered Spearman correlation matrix resulting from comparing the transcript expression values (TMM-normalized FPKM) for each pair of samples from both species: i.e. a) S. salar; b) D. labrax. [file 12864_2020_7317_MOESM12_ESM.docx]

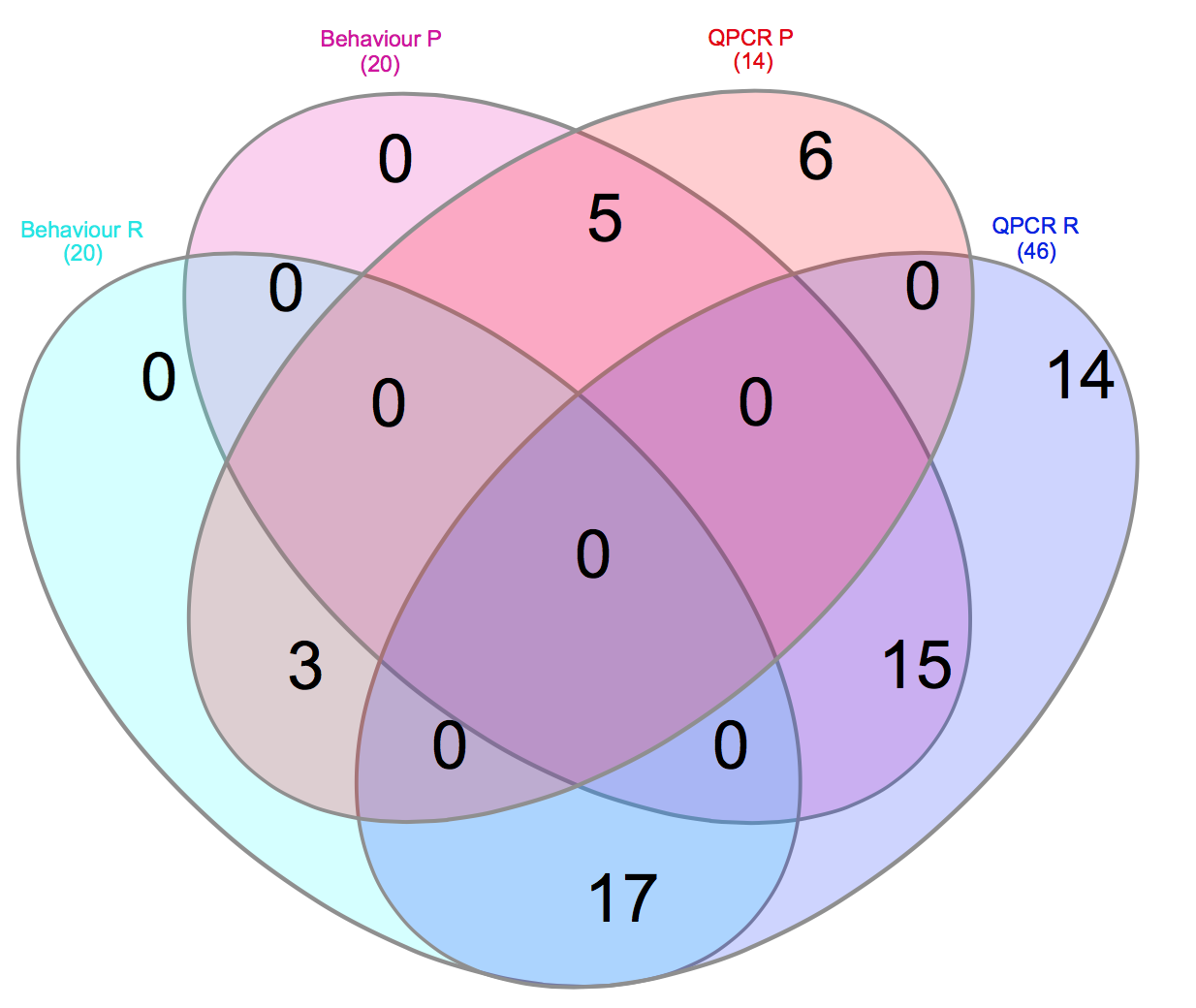

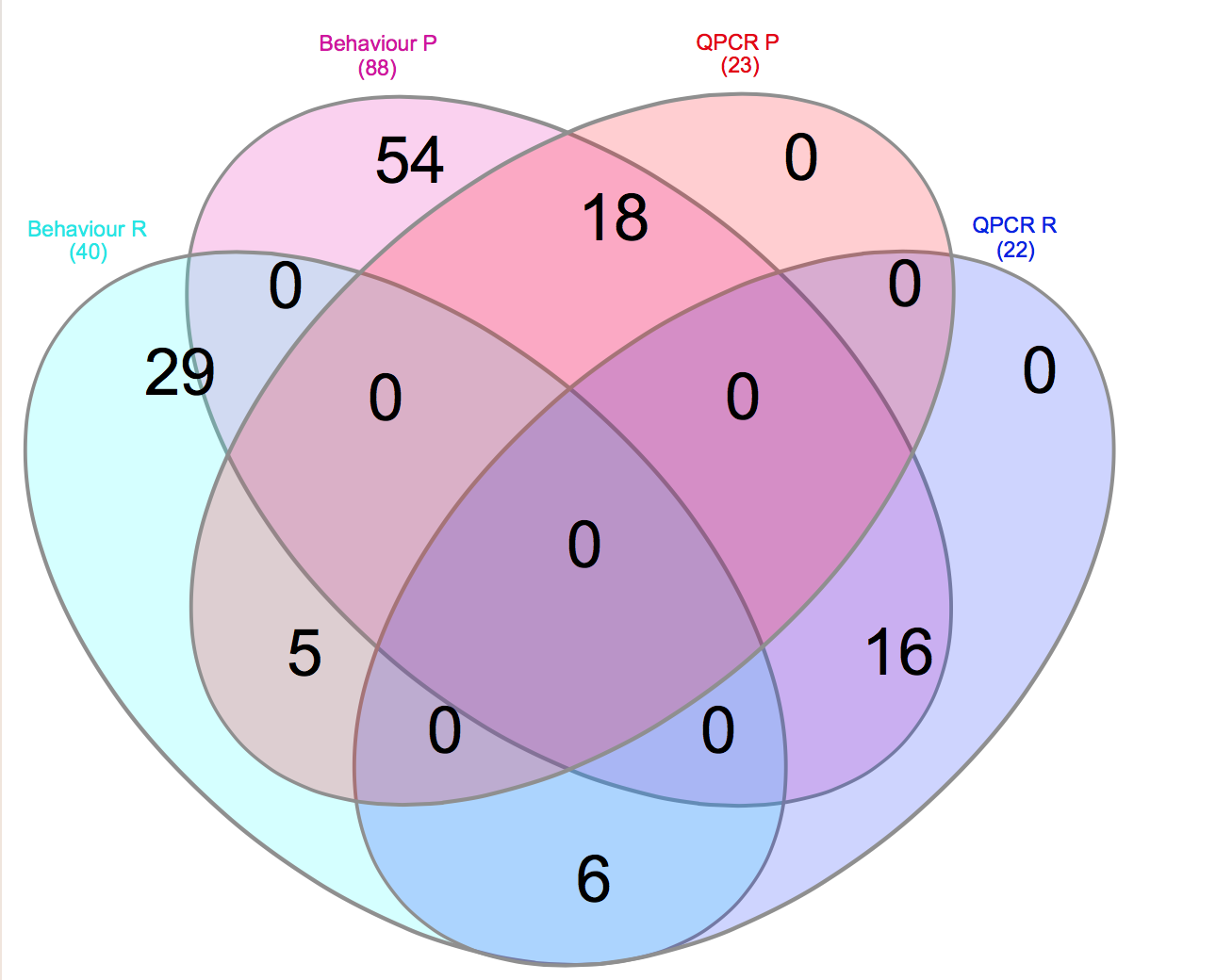


**b)**

**a)**

**Supporting_Figure1**. **Individual personality identification by both behavior test and QPCR assay**. Unique individual running number were used to identified proactive animals with both behavior boldness and behavior gene markers positive. a) S.salar proactive individuals N=18; b) D.labrax proactive individuals N= 5.

**Supporting_Figure2. Completeness estimation of Trinity assemblies of Illumina RNA-Seq by BUSCO.** Abbreviation: Ss: *S. salar*; Dl: *D. labrax*; Trans: transcriptome; Geno: Genome.

**a-ii)**

**a-i)**

**b-ii)**

**b-i)**

**Supporting_Figure3. Between-microarrays normalization of *D. rerio* brain transcriptome**. a) Distribution densities of probe intensities were compared before- (a-i) and after (a-ii) normalization; b) Log2-transformed intensities of each microarray were shown before before- (b-i) and after (b-ii) normalization.

**Supporting_Figure4. Transcriptomic gene list of D.rerio brain with annotations.** The numbers of annotated genes obtained by Microarray approach (Rey, Boltana et al. 2013) were shown in orange; while the ones obtained by Illumina RNA-Seq (Wong, McLeod et al. 2014) were shown in Dark Green.

**References**

Rey, S., S. Boltana, R. Vargas, N. Roher and S. MacKenzie (2013). "Combining animal personalities with transcriptomics resolves individual variation within a wild-type zebrafish population and identifies underpinning molecular differences in brain function." Molecular Ecology **22**(24): 6100-6115.

Wong, R. Y., M. M. McLeod and J. Godwin (2014). "Limited sex-biased neural gene expression patterns across strains in Zebrafish (Danio rerio)." BMC Genomics **15**(1): 1-9.

**b**

**a**

**Supporting_Figure 5. Spearman Correlation estimation of DEGs within each species sequenced by Illumina platform.**

Heat map showing the hierarchically clustered Spearman correlation matrix resulting from comparing the transcript expression values (TMM-normalized FPKM) for each pair of samples from both species: i.e. a) *S. salar*; b) *D. labrax*.
